# Comparison of Three-Bag Method Acetylcysteine Versus Two-Bag Method Acetylcysteine for the Treatment of Acetaminophen Toxicity: An Updated Systematic Review and Meta-Analysis

**DOI:** 10.3390/diseases12120332

**Published:** 2024-12-18

**Authors:** Mohammed Alrashed, Abdulrahman Alyousef, Hisham A. Badreldin, Khalid Bin Saleh, Shmeylan Al Harbi, Abdulkareem M. Albekairy, Abrar Alghamdi, Amal Al-Nahdi, Dhay Alonazi, Mohammed Alnuhait, Abdullah Alshammari, Tariq Alqahtani

**Affiliations:** 1 College of Pharmacy, King Saud bin Abdulaziz University for Health Sciences, Riyadh 14611, Saudi Arabia; alqrn1422@gmail.com (A.A.); aldeenh@ksau-hs.edu.sa (H.A.B.); salehk@ksau-hs.edu.sa (K.B.S.); harbish@ksau-hs.edu.sa (S.A.H.); bekairya@ksau-hs.edu.sa (A.M.A.); abrar.almrtan@gmail.com (A.A.); amalhamad.hn@gmail.com (A.A.-N.); ldhayll2@gmail.com (D.A.); qahtanita@ksau-hs.edu.sa (T.A.); 2 King Abdullah International Medical Research Center, Riyadh 11481, Saudi Arabia; 3 Pharmaceutical Care Department, King Abdulaziz Medical City, National Guard Health Affairs, Riyadh 11426, Saudi Arabia; 4 Department of Pharmacy Practice, College of Pharmacy, Umm Al-Qura University, Makkah 24382, Saudi Arabia; manuhait@uqu.edu.sa (M.A.); asshammari@uqu.edu.sa (A.A.)

**Keywords:** toxicity, acetaminophen, antidote

## Abstract

Background: Acetaminophen is generally considered safe when used according to the recommended guidelines. Consumption in excessive doses can lead to severe liver damage and, in critical cases, may even result in death. To reduce the effects of acetaminophen overdose, N-acetylcysteine (NAC) has been established as the preferred intervention to prevent liver damage. Objectives: The purpose of this updated systematic review and meta-analysis is to evaluate the potential benefits of a two-bag N-acetylcysteine (NAC) dosing regimen compared to the traditional three-bag protocol in the treatment of acetaminophen-induced liver toxicity. Methods: This systematic review was conducted in accordance with the Preferred Reporting Items for Systematic Reviews and Meta-Analyses guidelines. The research team utilized the PubMed and Cochrane databases to perform a thorough and comprehensive search of the relevant literature from the inception of these databases up until January 2024. Results: Nine studies were included. The overall use of two-bag NAC was associated with lower anaphylactic reactions and gastrointestinal symptoms compared to the three-bag method. The rate of liver toxicity resolution was the same between the two treatment groups. Conclusions: The two-bag NAC regimen can be considered a safe and effective method for managing acetaminophen toxicity.

## 1. Introduction

The World Health Organization (WHO) recognizes acetaminophen as one of the essential medications [1]. It is available over the counter (OTC) in most countries due to its safety profile for adults, children, and pregnant women. The recommended dose for adults and children aged 12 years and above is 500–1000 milligrams every 4–6 h as needed, with a daily maximum of 4 g. For children under 12 years, the recommended dosage is 15 mg per kilogram (kg) every 4–6 h as needed, up to a maximum of four doses per day [2]. Adult acetaminophen toxicity is associated with a single ingestion of 200 mg/kg or chronic ingestion of 150 mg/kg/day for 2 or more days. Annually, about 78,414 emergency department visits in the United States are attributed to non-abusive acetaminophen toxicity [3,4]. Pediatric toxicity is defined as a single dose > 200 mg/kg or chronic ingestion of ≥200 mg/kg over 24 h, 150 mg/kg/day over 48 h, or 100 mg/kg/day over 72 h or more. N-acetylcysteine (NAC) is the primary treatment for preventing liver injury following an acetaminophen overdose [4]. NAC replenishes glutathione stores and detoxifies the reactive metabolite N-acetyl-p-benzoquinone imine (NAPQI). Additionally, NAC has a wide therapeutic window, a shorter duration of action, and functions as a specific antidote, reducing the risk of cumulative toxicity. Traditionally, NAC is administered as a three-bag regimen: 150 mg/kg over 1 h, followed by 50 mg/kg over 4 h, and finally, 100 mg/kg over 16 h [5]. While the three-bag approach is effective in preventing liver injury, this approach poses complexities for healthcare providers, leading to potential administration errors. Additionally, the traditional regimen has been linked to anaphylactic reactions and fluid overload in patients [6,7]. Despite the three-bag regimen being the standard treatment for acetaminophen toxicity, evidence suggests that a two-bag regimen may be a simpler alternative [8,9,10,11]. The two-bag regimen consists of 200 mg/kg intravenous (IV) over 4 h, followed by 100 mg/kg IV over 16 h [12]. Therefore, systematic reviews and meta-analyses are necessary to compare the efficacy and safety of the three-bag versus the two-bag N-acetylcysteine regimen. While previous studies have explored the management of acetaminophen toxicity, this study provides a detailed synthesis of the published literature, focusing on simplifying treatment protocols and minimizing associated risks, thereby addressing critical gaps in the current clinical management of acetaminophen toxicity.

## 2. Materials and Methods

### 2.1. Search Strategy

The research team adhered to the Preferred Reporting Items for Systematic Reviews and Meta-Analyses (PRISMA) guidelines to conduct this systematic review. A comprehensive review was carried out using the PubMed, Embase, and Cochrane databases from their inception until January 2024, imposing no time or language restrictions [13]. The protocol for the systematic review of our study was not registered for PROSPERO.

### 2.2. Study Selection Criteria

The primary investigator set the study’s objectives and outlined the selection criteria to the research team members. The eligibility criteria were defined using the Population, Intervention, Comparator, and Outcomes (PICO) framework. The search process utilized pre-identified headings and keywords, including acetaminophen toxicity OR acetaminophen poisoning and N-acetyecysteine OR NAC. Studies were considered eligible for inclusion if they met the following criteria: (1) randomized controlled trials, prospective, or retrospective observational studies; (2) studies that compared three-bag N-acetylcysteine versus two-bag N-acetylcysteine; and (3) if the study evaluated at least one of the primary or secondary outcomes for this systematic review, as outlined in Section 2.3. The screening process consisted of two steps: the first one was to screen all data using pre-identified headlines and keywords, which were later verified by another senior member of the research team for proper adjudication. The references of the included studies were also manually searched.

### 2.3. Outcome Measures

Four reviewers evaluated effectiveness and safety outcome measures, extracting data using a standardized form. Primary outcomes included anaphylaxis reactions as defined by the original authors. Secondary outcomes involved gastrointestinal symptoms and resolution rate of liver toxicity.

### 2.4. Assessment of Study Quality and Publication Bias

The research team assessed the risk of bias for each study using the Cochrane Collaboration’s Risk of Bias Assessment Tool 2.0 for randomized controlled trials (RCTs). The Newcastle–Ottawa Scale (NOS) was used to evaluate the quality of observational cohort studies included in the analysis [14]. The NOS allocates up to nine stars for observational cohort studies: four stars for the selection of participants, two stars for the comparability of cohorts based on design and analysis, and three stars for the assessment of outcomes and adequacy of follow-up. The quality of the included observational cohort studies was categorized based on the number of stars awarded: low quality (0 to 3 stars), medium quality (4 to 6 stars), and high quality (7 to 9 stars). Three authors independently assessed the quality of the included studies. In cases of disagreement, a fourth experienced author was consulted to reach a consensus. Data collected from each study included the following: study authors, year of publication, study design, N-acetylcysteine regimen and dosage, number of patients in both the treatment and control groups, duration of follow-up, and clinical outcomes of interest.

### 2.5. Data Synthesis and Statistical Analysis

The Mantel–Haenszel random effects model was utilized to calculate risk ratios (RRs) and corresponding 95% confidence intervals (CIs) to estimate the pooled treatment effects of the 3-bag and 2-bag doses of N-acetylcysteine (NAC) for emergency acetaminophen reversal. This model was selected due to the variability in patient populations and clinical outcomes observed across the included studies. Study heterogeneity was evaluated using Cochrane’s Q test and the I^2^ statistic, with I^2^ values of <25% indicating low heterogeneity, 25–50% indicating moderate heterogeneity, and >75% indicating high heterogeneity. A *p*-value of <0.1 from the Cochrane Q test was considered indicative of significant heterogeneity. Publication bias was assessed through funnel plots. All statistical analyses were performed using the standard statistical procedures of RevMan 5.2 [15]

## 3. Results

### 3.1. Included Studies

**Figure 1:**
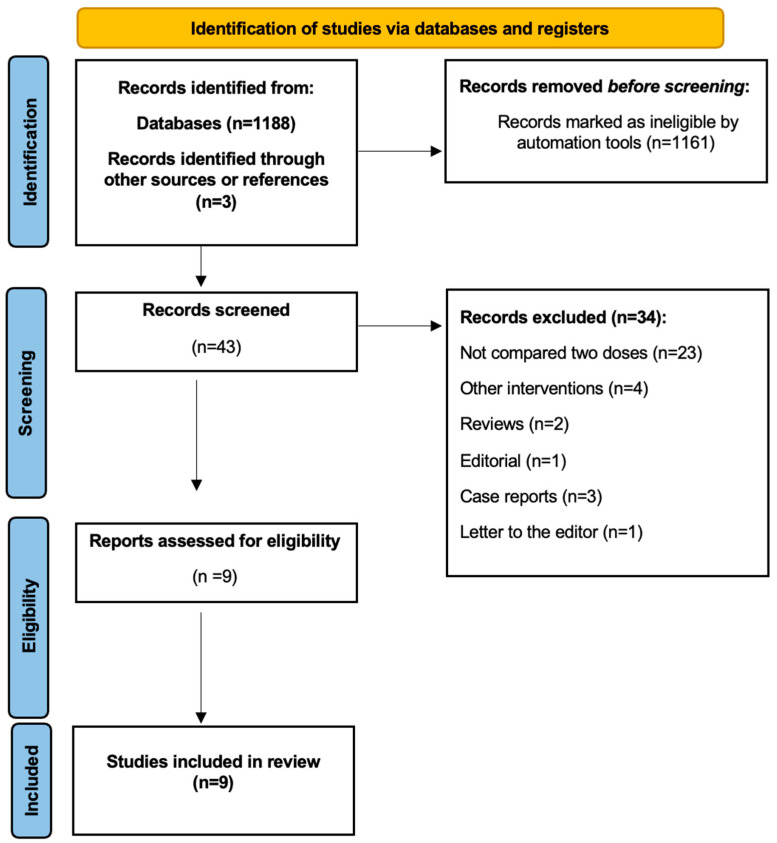
Preferred Reporting Items for Systematic Reviews and Meta-Analyses diagram indicating the method for selection of papers included in the present study.

The literature search yielded 1188 potential studies from the database, and an additional three records were identified manually through references. Of these, 1161 studies were excluded based on an initial screening. The remaining 43 studies underwent further screening for eligibility. A significant number of these studies were excluded because they either did not compare the two dosages or utilized interventions. Nine studies met our inclusion criteria and were subsequently included in our analysis. A flowchart outlining the literature search and selection process for the studies is presented in Figure 1.

### 3.2. Study Characteristics and Quality Assessment

Nine studies met the eligibility criteria. The characteristics of the identified studies are summarized in Table 1. Most of the studies were either retrospective or prospective observational in nature. All eligible cohort studies demonstrated medium quality with mean NOS scores ranging from 3 to 6 (Table 1). Each study aimed to compare the efficacy and safety of three-bag versus two-bag NAC for the reversal of emergent acetaminophen toxicity. For the meta-analysis, data across nine studies were analyzed. This included 7767 patients treated with NAC therapy. Among the studies included, only one was an observational prospective study, one was a randomized controlled trial, and the remaining seven were observational retrospective studies. Each observational study included a comparison with a control group.

### 3.3. Results of the Meta-Analysis

#### 3.3.1. Anaphylactic Reactions

**Figure 2:**
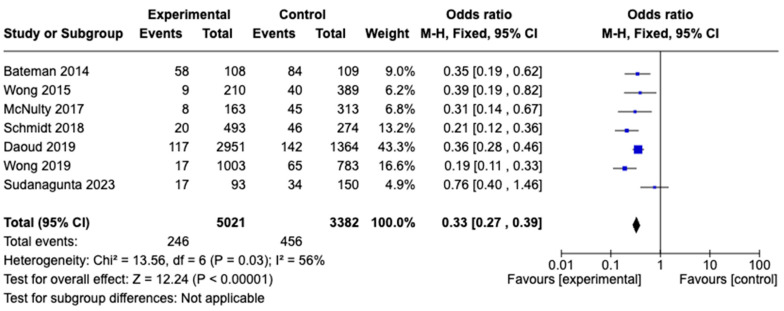
Meta-analysis comparing the incidence of anaphylactoid reactions between patients treated with the two-bag and three-bag NAC regimens ([9,12,17,18,19,20,23]).

Anaphylactoid reactions were reported in seven studies, with the pooled rate evaluated from data on 8403 patients (three-bag dosing, n = 5021; two-bag dosing, n = 3382). The studies employed various criteria to assess anaphylactoid reactions. The most-used definitions described a severe, life-threatening, generalized, or systemic hypersensitivity reaction involving the airway and/or breathing and/or circulation. The proportion of patients who had anaphylactic reactions did significantly differ between the three-bag and two-bag dosing strategies (Odds Ratio [OD], 0.33; 95% CI, 0.27–0.39; *p* = 0.03). Overall, the studies exhibited moderate to high heterogeneity (I^2^ = 56%). A forest plot analysis depicting these data is presented in Figure 2.

#### 3.3.2. Gastrointestinal Symptoms

**Figure 3:**
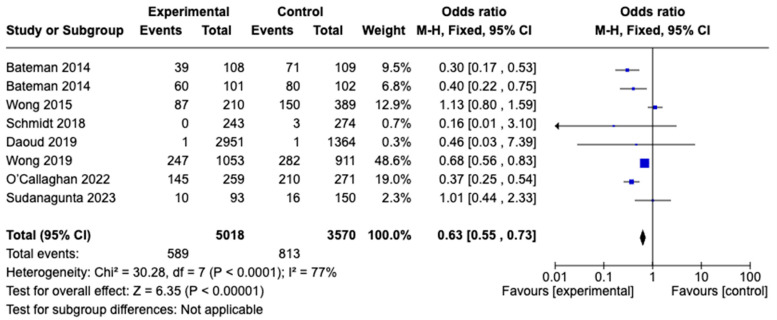
Meta-analysis of the incidence of gastrointestinal symptoms with the two-bag NAC regimen versus the three-bag regimen ([12,17,18,19,20,23]).

Gastrointestinal symptoms were reported in eight studies, with the pooled rate evaluated from data on 4435 patients (three-bag dosing, n = 2304; two-bag dosing, n = 2131). The studies employed various criteria to assess gastrointestinal symptoms. The most-used definitions included nausea, vomiting, or both. The proportion of patients who had gastrointestinal symptoms did significantly differ between the three-bag and two-bag dosing strategies (Odds Ratio [OD], 0.63; 95% CI, 0.55–0.73; *p* < 0.05). Overall, the studies exhibited high heterogeneity (I^2^ = 77%). A forest plot analysis depicting these data is presented in Figure 3.

#### 3.3.3. Liver Toxicity

**Figure 4:**
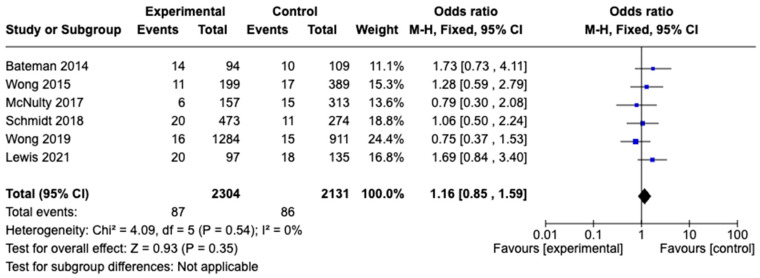
Meta-analysis of the resolution rate of hepatotoxicity with the two-bag NAC regimen versus the three-bag regimen ([9,12,17,18,19,21]).

The rate of liver injury resolution was reported in six studies, with the pooled rate evaluated from data on 8588 patients (three-bag dosing, n = 5018; two-bag dosing, n = 3570). The studies employed various criteria to assess the rate of liver toxicity. The most-used definitions were ALT > 1000IU/L. The proportion of patients with liver toxicity resolution did not significantly differ between the three-bag and two-bag dosing strategies (Odds Ratio [OD], 1.16; 95% CI, 0.85–1.59; *p* = 0.54). Overall, the studies exhibited low heterogeneity (I^2^ = 0%). A forest plot analysis depicting these data is presented in Figure 4.

## 4. Discussion

This systematic review and meta-analysis provide critical insights into the efficacy and safety of two different NAC regimens in the management of acetaminophen toxicity; it reveals critical insights into optimizing treatment protocols to reduce adverse reactions while maintaining effectiveness against hepatotoxicity. Our study addressed the use of variable NAC doses (both two-bag and three-bag dosing strategies) for acetaminophen toxicity. Nine studies with 7767 patients treated with the three-bag method and 4068 with two-bag NAC therapy were identified for reversing acetaminophen toxicity. We observed a standardized method of NAC administration; patients in the two-bag group were more likely to receive 200 mg/kg over 4 h followed by 100 mg/kg over 16 h compared to the three-bag group, which primarily followed the package insert recommendations [23]. Studies reported a consistent finding that hepatotoxicity rates did not significantly differ between traditional and modified NAC regimens [17,18,19]. These trials show that shorter or two-bag regimens can effectively prevent severe liver damage as effectively as the longer three-bag regimen. Lewis et al. further support this, showing no significant difference in hepatotoxicity, which implies that a shorter duration of NAC treatment may be sufficient for patients with massive acetaminophen overdoses when administered in a timely manner. A routine implementation of a three-bag dosing strategy in emergency departments is not justified and could delay NAC administration [20].

A significant advantage of modified regimens, particularly the two-bag system, is the reduction in adverse reactions. Studies have highlighted a lower incidence of adverse events, such as anaphylactoid reactions and vomiting, among patients treated with the two-bag method compared to three-bag NAC therapy [17,18,21,22,23]. This reduction not only enhances patient outcomes but also potentially increases adherence to treatment protocols in emergency settings. The three-bag regimen includes a high-dose bolus in the first infusion, which likely results in a rapid peak in plasma NAC concentration. This abrupt increase may contribute to the higher incidence of anaphylactoid reactions observed with the three-bag approach. These findings support the conclusions of our study, recommending the two-bag NAC regimen as the preferred initial therapy for acetaminophen toxicity.

The modified protocols also contribute to reducing medication errors and hospital stay durations. Sudanagunta et al. found that shorter stays and fewer medication errors associated with two-bag regimens suggest improvements in healthcare efficiency and patient safety [20]. Such changes not only enhance patient outcomes but also reduce healthcare costs and resource utilization, crucial in high-volume settings like emergency departments. The collected data from these studies advocate for a shift towards simplified NAC regimens in clinical practice, particularly for patients presenting early and with low to moderate risk of severe hepatotoxicity. However, the decision to implement a shorter or two-bag regimen should be carefully considered based on individual patient risk assessments, including the timing of overdose and initial liver function tests. Although the standard regimen in practice is still the three-bag approach, we can expect that there will be a change in the future to the two-bag approach, especially if a randomized clinical trial with a large sample size and adequate follow-up is performed. Simplifying the administration of acetylcysteine could also provide several advantages that have not been tested in this systematic review and meta-analysis including simplifying the administration of the antidote by nurses. Furthermore, administration of acetylcysteine using the two-bag approach may reduce administration errors and reduce the risk of volume overload in patients, particularly patients with comorbidities such as heart failure and chronic kidney disease [6,7].

### Strengths and Limitations

This meta-analysis and systematic review represent the most recent comprehensive assessment of two-bag versus three-bag NAC administration in patients with acetaminophen toxicity. Our approach aimed to provide a practical evaluation of two-bag versus three-bag NAC use, considering the various clinical scenarios and their implications. While acknowledging the strengths of the study, we also recognize several limitations. Most of the included studies were either retrospective or prospective, which could potentially influence the robustness of our findings. Variations in reporting and the emergent nature of interventions limited our ability to evaluate differences in clinical management or alternative treatments among the studies. We refrained from assessing the correlation between post-administration changes in liver function tests due to inconsistent reporting across the included studies. Additionally, the significant variability between the two-bag and three-bag NAC dosing regimens across studies, including differences in initial bolus doses, infusion durations, and total NAC exposure, introduces bias and complicates direct comparisons. This lack of standardization reflects the heterogeneity in clinical practice and may affect treatment outcomes, emphasizing the need for future research with uniform dosing protocols to ensure more reliable comparisons. The study did not uniformly account for important factors such as concurrent medication use and patients’ medical history, which are crucial in influencing initial therapy decisions in the ED. This oversight could lead to variations in treatment efficacy and patient outcomes. The failure to consistently integrate these factors into the analysis might have introduced biases or confounding variables, thus impacting the reliability and applicability of the findings. This limitation highlights the need for more detailed data collection and consideration of individual patient profiles in future research to enhance the precision of treatment recommendations and optimize patient care in emergency settings. The study was unable to control for publication bias or ensure complete homogeneity across the included studies, which might have affected the overall analysis. Furthermore, the differences in study designs, populations, and assessment criteria among the included studies add complexity when attempting to draw definitive conclusions. The significant heterogeneity, with I^2^ values of 56% for both anaphylactic reactions and gastrointestinal (GI) symptoms, underscores the variability among the studies. These I^2^ values, which are both above the 50% threshold, suggest substantial inconsistency in study outcomes. Further research should aim to standardize study designs and definitions to reduce heterogeneity and improve the reliability of conclusions in future meta-analyses. In summary, while our study offers valuable insights into the comparative effectiveness of different NAC dosing strategies, these findings are constrained by the limitations inherent in the diverse nature and reporting standards of the included studies. Further research that considers these limitations is essential to gain a more comprehensive understanding of optimal NAC dosing in acetaminophen toxicity scenarios.

## 5. Conclusions

In summary, this comprehensive review indicates that two-bag and abbreviated NAC regimens provide a promising approach for the treatment of acetaminophen overdose, offering safety, efficacy, and improved patient compliance compared to the traditional three-bag regimen. Given these benefits, it is recommended that healthcare providers evaluate the potential for integrating these protocols into practice, which could lead to enhanced patient management and more efficient use of medical resources. However, the findings should be interpreted with caution due to the limited number of high-quality clinical trials included in this review. Future research with standardized protocols and larger sample sizes is essential to validate these conclusions and strengthen the evidence base.

## Figures and Tables

**Table 1:** Characteristics of the studies included in the meta-analysis.

#	Author, Year	Study Design	N-Acetylcysteine Dose	Number of Patients	NOS
1	Bateman, 2014 [16]	RCT	Two-bag regimen 100 mg/kg in 200 mL, over 2 h 200 mg/kg in 1 L, over 10 h	108 patients	5
			Three-bag regimen 150 mg/kg in 200 mL, over 15 min 50 mg/kg in 0.5 L, over 4 h 100 mg/kg in 1 L, over 16 h	109 patients	
2	Wong, 2015 [12]	Retrospective	Two-bag regimen 200 mg/kg over 4 h, followed by 100 mg/kg over 16 h	210 patients	5
			Three-bag regimen 150 mg/kg over 1 h, 50 mg/kg over 4 h, and 100 mg/kg over 16 h	389 patients	
3	McNulty, 2017 [9]	Prospective	Two-bag regimen 200 mg/kg over 4 h, followed by 100 mg/kg over 16 h	163 patients	5
			Three-bag regimen 150 mg/kg over 1 h, 50 mg/kg over 4 h, and 100 mg/kg over 16 h	313 patients	
4	Wong, 2018 [17]	Retrospective	Two-bag regimen 200 mg/kg over 4 h, followed by 100 mg/kg over 16 h	8 patients	4
			Three-bag regimen 150 mg/kg over 1 h, 50 mg/kg over 4 h, and 100 mg/kg over 16 h	21 patients	
5	Schmidt, 2018 [18]	Retrospective	Two-bag regimen 200 mg/kg over 4 h, followed by 100 mg/kg over 16 h	493 patients	6
			Three-bag regimen 150 mg/kg over 1 h, 50 mg/kg over 4 h, and 100 mg/kg over 16 h	274 patients	
6	Daoud, 2019 [19]	Retrospective	Two-bag regimen 200 mg/kg over 4 h, followed by 100 mg/kg over 16 h	2951 patients	5
			Three-bag regimen 150 mg/kg over 1 h, 50 mg/kg over 4 h, and 100 mg/kg over 16 h	1364 patients	
7	Lewis, 2019 [20]	Retrospective	Standard-dose IV NAC: No specification	135 patients	3
			High-dose IV NAC: 150 mg/kg IV loading dose, followed by 50 mg/kg infusion over 4 h, followed by 200 mg/kg infusion over 16 h	117 patients	
8	O’Callaghan, 2022 [21]	Retrospective	Two-bag regimen 200 mg/kg over 4 h, followed by 100 mg/kg over 16 h	598 patients	4
			Three-bag regimen 150 mg/kg over 1 h, 50 mg/kg over 4 h, and 100 mg/kg over 16 h	271 patients	
9	Sudanagunta 2023 [22]	Retrospective	Two-bag regimen 200 mg/kg over 4 h, followed by 100 mg/kg over 16 h	93 patients	5
			Three-bag regimen 150 mg/kg over 1 h, 50 mg/kg over 4 h, and 100 mg/kg over 16 h	150 patients	

## Data Availability

No new data were created or analyzed in this study. The data used for the systematic review were obtained from previously published studies, which are cited within this article.

## References

[1] World Health Organization (2021). WHO Model List of Essential Medicines—22nd List, 2021.

[2] Therapeutic Goods Administration (TGA) (2023). Recommended Paracetamol Doses.

[3] Budnitz D.S., Lovegrove M.C., Crosby A.E. (2011). Emergency Department Visits for Overdoses of Acetaminophen-Containing Products. Am. J. Prev. Med..

[4] Fisher E.S., Curry S.C. (2019). Evaluation and Treatment of Acetaminophen Toxicity. Adv. Pharmacol..

[5] Ershad M., Naji A., Vearrier D. (2019). N-Acetylcysteine. Adv. Pharmacol..

[6] Hayes B.D., Klein-Schwartz W., Doyon S. (2008). Frequency of Medication Errors with Intravenous Acetylcysteine for Acetaminophen Overdose. Ann. Pharmacother..

[7] Institute for Safe Medication Practices Canada (2023). Preventing Errors with Intravenous Acetylcysteine. ISMP Can. Saf. Bull..

[8] Cole J.B., Thomas M., Zhang W. (2023). Is Two Better Than Three? A Systematic Review of Two-Bag Intravenous N-Acetylcysteine Regimens for Acetaminophen Poisoning. West. J. Emerg. Med..

[9] McNulty R., Lim J.M.E., Chandru P., Gunja N. (2017). Fewer Adverse Effects with a Modified Two-Bag Acetylcysteine Protocol in Paracetamol Overdose. Clin. Toxicol..

[10] Alrossies A.S. (2022). Evaluation of a Shorter 12h Acetylcysteine Regimen and Development of a Simpler Acetylcysteine (SNAP) Protocol for the Treatment of Paracetamol Poisoning. Doctoral Dissertation.

[11] Johnson M.T., Brewer K.L., Young R.S. (2011). Evaluation of a Simplified N-Acetylcysteine Dosing Regimen for the Treatment of Acetaminophen Toxicity. Ann. Pharmacother..

[12] Wong A., Graudins A. (2016). Simplification of the Standard Three-Bag Intravenous Acetylcysteine Regimen for Paracetamol Poisoning Results in a Lower Incidence of Adverse Drug Reactions. Clin. Toxicol..

[13] Page M.J., McKenzie J.E., Bossuyt P.M., Boutron I., Hoffmann T.C., Mulrow C.D., Shamseer L., Tetzlaff J.M., Aki E.A., Brennan S.E. (2021). The PRISMA 2020 Statement: An Updated Guideline for Reporting Systematic Reviews. BMJ.

[14] Wells G.A., Shea B., O’Connell D., Peterson J., Welch V., Losos M., Tugwell P. The Newcastle-Ottawa Scale (NOS) for Assessing the Quality of Nonrandomized Studies in Meta-Analyses. http://www.ohri.ca/programs/clinical_epidemiology/oxford.asp.

[15] Review Manager (RevMan) (2012). Computer Program, Version 5.2.

[16] Bateman D.N., Dear J.W., Thanacoody H.K.R., Thomas S.H.L. (2014). Effectiveness of the Modified Two-Bag Acetylcysteine Protocol for the Treatment of Paracetamol Poisoning. BMJ.

[17] Wong A., Homer N., Dear J.W., Choy K.W., Doery J., Graudins A. (2018). Paracetamol Metabolite Concentrations Following Low-Risk Overdose Treated with an Abbreviated 12-h Versus 20-h Acetylcysteine Infusion. Clin. Toxicol..

[18] Schmidt L.E., Rasmussen D.N., Petersen T.S., Macias-Perez I.M., Pavliv L., Kaelin B., Dart R.C., Dalhoff K. (2018). Fewer Adverse Effects Associated with a Modified Two-Bag Intravenous Acetylcysteine Protocol Compared to Traditional Three-Bag Regimen in Paracetamol Overdose. Clin. Toxicol..

[19] Daoud A., Dalhoff K.P., Christensen M.B., Bøgevig S., Petersen T.S. (2019). Two-Bag Intravenous N-Acetylcysteine, Antihistamine Pretreatment, and High Plasma Paracetamol Levels Are Associated with a Lower Incidence of Anaphylactoid Reactions to N-Acetylcysteine. Clin. Toxicol..

[20] Lewis J.C., James R., Young R. (2021). High-Dose N-Acetylcysteine Treatment Does Not Affect the Odds of Hepatotoxicity in Acute Massive Acetaminophen Overdose. J. Clin. Pharmacol..

[21] O’Callaghan C., Graudins A., Wong A. (2022). A Two-Bag Acetylcysteine Regimen Is Associated with Shorter Delays and Interruptions in the Treatment of Paracetamol Overdose. Clin. Toxicol..

[22] Sudanagunta S., Camarena-Michel A., Pennington S., Leonard J., Hoyte C., Wang G.S. (2022). Comparison of Two-Bag Versus Three-Bag N-Acetylcysteine Regimens for Pediatric Acetaminophen Toxicity. Ann. Pharmacother..

[23] Cumberland Pharmaceuticals Inc. Acetadote^®^ (Acetylcysteine) Injection [Package Insert]. NDA 21-539/S-004, 2006. https://www.accessdata.fda.gov/drugsatfda_docs/label/2006/021539s004lbl.pdf.

